# Harnessing digital footprint data for population health: a discussion on collaboration, challenges and opportunities in the UK

**DOI:** 10.1136/bmjhci-2024-101119

**Published:** 2024-09-28

**Authors:** Romana Burgess, Elizabeth Dolan, Neo Poon, Victoria Jenneson, Francesca Pontin, Torty Sivill, Michelle Morris, Anya Skatova

**Affiliations:** 1 MRC Integrative Epidemiology Unit, Bristol, UK; 2 Digital Footprints Lab, Univeristy of Bristol, Bristol, UK; 3 Nottingham Business School, University of Nottingham, Nottingham, UK; 4 University of Leeds, Leeds Institute for Data Analytics, Leeds, UK; 5 University of Leeds School of Food Science and Nutrition, Leeds, UK; 6 University of Leeds, Consumer Data Research Centre, Leeds, UK; 7 University of Bristol School of Computer Science Electrical and Electronic Engineering and Engineering Maths, Bristol, UK; 8 The Alan Turing Institute, London, UK

**Keywords:** Public Health, Data Science, Public health informatics

## Abstract

Digital footprint data are inspiring a new era in population health and well-being research. Linking these novel data with other datasets is critical for future research wishing to use these data for the public good. In order to succeed, successful collaboration among industry, academics and policy-makers is vital. Therefore, we discuss the benefits and obstacles for these stakeholder groups in using digital footprint data for research in the UK. We advocate for policy-makers’ inclusion in research efforts, stress the exceptional potential of digital footprint research to impact policy-making and explore the role of industry as data providers, with a focus on shared value, commercial sensitivity, resource requirements and streamlined processes. We underscore the importance of multidisciplinary approaches, consumer trust and ethical considerations in navigating methodological challenges and further call for increased public engagement to enhance societal acceptability. Finally, we discuss how to overcome methodological challenges, such as reproducibility and sharing of learnings, in future collaborations. By adopting a multiperspective approach to outlining the challenges of working with digital footprint data, our contribution helps to ensure that future research can navigate these challenges effectively while remaining reproducible, ethical and impactful.

## Introduction

 The data collected from online activities—known as digital footprint data (or ‘smart data’)—have emerged as a novel approach to exploring public health and well-being. Rich digital information is generated as we traverse our daily lives, leaving footprints of our behaviours; from financial well-being (eg, banking data) to eating habits (eg, food purchase records) and physical activity levels (eg, phone accelerometers). While traditionally used by businesses for marketing purposes, the potential for using large amounts of data to forecast population-level outcomes is of increasing relevance in policy-making. However, many stand-alone digital footprint data do not contain direct measures of health and well-being, thus, interest has turned to exploring novel data linkages.

Linking digital footprint data with other datasets—such as cohort studies, observational surveys or administrative data on education or health—offers innovative opportunities to use data for the public good. Each of these data sources carries its own biases. For instance, digital footprint data may be skewed towards individuals with higher technical competency while cohort studies may have selection biases related to retention rates. Yet despite individual biases, triangulating data sources via novel data linkages can mitigate some of these inherent limitations. By integrating multiple datasets, we can cross-validate and complement the data, leading to potentially more accurate and comprehensive insights. For instance, linking digital footprint data with health records can reveal patterns not visible in self-reported health surveys, providing more reliable prevalence statistics.

These linkages can also enable powerful evaluation of existing policy interventions or natural experiments resulting from real-world events (eg, changes in policy during the pandemic) that may inform future decision-making. At the individual level, linkage facilitates longitudinal research into cause and effect, for instance, by integrating digital footprint data into other longitudinal datasets such as cohort studies. This linkage allows researchers to track changes over time and identify causal relationships, for example, via historic records of health and well-being. At an area level, linkage allows observational research, associating national outcomes with individual behaviours.

Digital footprint data serve as proprietary information that can be used by researchers for public good, such as for improving the accuracy of respiratory disease forecasting models by integrating medication sales data,^1^ understanding mental health behaviours through social media use,^2^ or enhancing urban planning using mobility data.^3^ Linking digital footprint data to other data sources requires collaboration among diverse stakeholders, including consumers, industry, public institutions, academics, media, non-governmental organisations and policy-makers. Due to their pivotal roles in research, data sharing and shaping data linkage initiatives, this discussion primarily focuses on industry, academics and policy-makers. The challenge lies in fostering cohesive efforts among these three stakeholders.

To further this aim, we established the Turing Novel Data Linkages Special Interest Group (see online supplemental appendix^4^,^7^), which brings together industry experts, academics and policy-makers to collaboratively work with these data. As such, based on the multiple perspectives represented by this group, this discussion aims to:

1. Identify the benefits and obstacles unique to each of these stakeholder groups, and;
2. Propose effective solutions to address these challenges in the future.

### Novel digital footprint data linkages can benefit policy-makers

Policy-maker involvement in digital footprint research is vital for achieving impactful outcomes. Their influence through laws, political pressure and extensive networks makes them essential stakeholders for data linkage, and without their engagement, delivering meaningful impact is hindered. Policy-makers play a vital role in ensuring the necessary legal frameworks and political support are in place to facilitate the effective use of data, and as such, we must continue to engage these key stakeholders in the conversation going forward.

Linked digital footprint datasets can inform policy design, for instance, by revealing lifestyle precursors of health outcomes to guide preventative interventions. This calls for research programmes with extended time frames—demanding substantial support from funding bodies—covering data source identification, data linkage and policy intervention crafting. The linked data can not only provide evidence to develop interventions but also serves as tools to measure their impact on health, lifestyle and individual well-being.

Analysing linked datasets can also bridge regional and national policies since statistical inference with geospatial data can be made on both aggregated and individual levels. There is a need for better support and alignment of bottom-up (from local interventions to national policies) and top-down research (from nationwide datasets to local areas). For example, focusing on a specific area (eg, Bradford)^8^ for interventions can expedite insights, which can later be applied to nationwide analyses using countrywide datasets. We can then study regional differences in health and well-being to uncover why those differences exist. In this way, improving infrastructure for novel data linkages within existing cohort studies (eg, Born-in-Bradford and Avon Longitudinal Study of Parents and Children)^9 10^ is an obvious place to innovate.

### Exploring industry’s incentives and obstacles to share data for the public benefit is imperative

Effective industry collaboration in digital footprint research is essential for producing valuable insights that serve both public interests and organisational goals. This can ensure shared value between businesses, academics and consumers, enhancing research outputs and business practices while contributing to broader societal advancements. For example, working towards carbon net zero has clear planetary benefits, as well as reputational and potential cost-saving benefits for businesses.

Current data-sharing practices are often inefficient and impractical, typically requiring collaboration from many points of contact within data holder organisations (ie, within industry). To enable more agile and effective data sharing, we must implement a common, streamlined and yet flexible process that can be customised to address major industry priorities. Standardised data-sharing agreements, centralised data repositories and employing data anonymisation techniques could reduce legal and administrative burdens while ensuring privacy. However, these processes can be hard to standardise as different companies have unique priorities, methods and sensitivities regarding data sharing. Customisable frameworks and adaptable solutions may, therefore, be necessary to accommodate these variations.

As the prerequisites of data sharing range from ethical reviews to data governance sign-offs—formal processes which ensure compliance with legal, ethical and security standards, including data privacy protection—and technical capabilities, training within industry partner organisations can support the entire data linkage process. There is a need to build capacity inside businesses around the data management pipeline, in addition to ethical and legal considerations. In the short term, this could be done through training existing staff; in the longer term, it is through teaching these skills to doctoral candidates and students who will become the future workforce.

Pragmatic solutions for sharing data—considering the UK General Data Protection Regulation (GDPR) and other data governance issues—should be developed. The cost for the industry to share data with researchers is often prohibitive, involving expensive data-extract queries, staff time allocation and resource-consuming legal agreements. Budgets and timescales of research grants and businesses must address these resource requirements. The COVID-19 pandemic serves as a prominent example where businesses absorbed the costs of data sharing and linkage, gaining important additional perspectives from their data. Continual feedback loops and analytic insights are consequently considered to be the way forward, as they provide businesses with updates on data use and benefits, revealing actionable insights they might not identify independently. By seeing tangible outcomes from their shared data, businesses are incentivised to continue and expand their data-sharing practices.

Commercial sensitivity poses a significant challenge to data sharing from the industry perspective. This includes potential sensitivity in product supply chain data, which can reveal details about suppliers and contracts. Even identifying a specific problem that the data project might address can be commercially sensitive; for instance, if an output establishes that those purchasing food high in sugar in shop X are at higher risk of diabetes while the conclusion is generalisable to all stores, this may be misrepresented by the media as an issue specific to shop X. Stakeholders must consider sensitivity from the onset, and discussing best practices in sharing both commercially sensitive and non-sensitive data with industry representatives is crucial. Additionally, addressing potential conflicts between academic and industry interests from data-derived learnings (eg, intellectual property rights) necessitates good practice guides and dedicated support within universities.

### Successful past linkages can provide vital insights

Health and well-being is an important application for digital footprint research. Real-world physical and mental health data far exceed the data collated by doctors in our medical records; digital footprint data can help us to understand how lifestyle and consumer behaviours can be used as markers of health and well-being, determinants of risk factors for disease and proxies of inequalities. In addition, a geodemographic approach to data aggregation and linkage provides valuable insights into understanding how health patterns vary spatially, but individual linkage will be indispensable for unlocking more meaningful insights.

A stark example of this exists for cancer: while the main outcome in health records is typically binary, indicating whether a patient is alive or deceased, this does not provide a comprehensive picture of their quality of life or overall well-being. By analysing digital footprint data, like online activity or supermarket transactions, we can gain insights into various aspects of a cancer patient’s life, such as their emotional state, social interactions, treatment adherence and symptom development. For example, monitoring over-the-counter pain and indigestion medication purchases could potentially indicate early symptoms of ovarian cancer.^11^

Numerous examples of successful novel data linkages exist, including linking loyalty card transactions to survey data to investigate whether purchase records represent self-reported food consumption,^12^ and connecting health and education data to reveal socioeconomic variation in reading glasses prescription uptake, prompting local interventions.^13^ While examples like the National Programme for IT and care data highlight opportunities for improvement,^14^ other case studies serve to highlight the potential misuse of linked data (eg, tracking hormone cycles data violating female reproductive rights^15 16^ or influencing behaviour via targeted marketing in commercial settings).

Yet, sharing successful case studies will be extremely valuable to this community. These should consider multiple stakeholder perspectives, identify clear expectations of how data will be used and avoid overgeneralisation—as the specific details are important. Determining a recommended set of dissemination outputs from novel data linkages is valuable and will allow us to establish the standard in this approach to scientific investigation. This may include an academic paper, published code, open anonymised and/or aggregated datasets, policy reports and industry reports. Success stories should be publicly accessible and made available to all audiences, through presentations at workshops or videos/blogs on dedicated websites.

### Consumers should be prioritised at every stage of the research

As the producers and oftentimes owners of the data, consumer participation is vital to ensure that research aligns with public values while also promoting accountability and transparency among researchers. As such, consumers should be consulted and engaged with frequently on data usage, privacy concerns and consent processes, among other aspects.

Communicating research to the public requires framing in terms of individual concerns and emphasising the intended outcomes (eg, a better understanding of early disease symptoms). The bidirectional nature of public engagement ensures that consumer opinion is fed back to researchers and industry, with the power of individual quotes that represent people’s viewpoints being a useful tool to obtain an overview of public acceptability and understanding. Feedback from consumers can encourage both researchers and the industry to fine-tune the parameters of data sharing.

Further, it is important to consider how the public perceives the motives of businesses sharing data—beyond presumed financial incentive—and improve their understanding of the value of digital footprint research. Ensuring consumer acceptability requires presenting risks alongside benefits and providing control over data usage. Public opinion on novel data linkages aligns with corporate social responsibility commitments and Environmental Social and Corporate Governance, therefore, industry data holders could benefit from prioritising public awareness and conducting engagement work to highlight the utility of linked datasets for public good, explaining the safe use of data.

### Ethical considerations stand as a priority for all stakeholders

Ethics and governance are at the heart of novel data linkages. Ensuring that research adheres to relevant data protection laws (eg, GDPR in Europe) is vital for protecting individual privacy and harnessing data responsibly.

For participants, the implications of using their data are significant, which is why we must ensure that participants fully understand how their data will be used, shared and protected. Informed consent must be obtained, with clear explanations of any potential risks associated with the research. In addition, while voluntary data donation is an ethical framework for data collection,^17^ self-selection of participants could perpetuate biases and overlook minority groups, highlighting considerations regarding inclusivity. Additionally, researchers must prioritise the prevention of reidentification of anonymised individuals and adhere to data minimisation principles, collecting only the data necessary for the research at hand.

For academics, policy-makers and industry professionals, the practical implications of ethical data use encompass data storage, sharing and security. Trusted Research Environments are the gold standard for secure data analysis,^18^ ensuring that data ownership and access control are clearly defined, preventing misuse and promoting ethical and responsible data usage. These environments also help to establish accountability mechanisms for potential data breaches. Further, novel data linkages challenge existing ethical frameworks within universities and the National Health Service, designed for traditional primary data collection. Early engagement to extend these frameworks is recommended. By proactively adapting ethical standards to accommodate the complexities of novel data linkages, we can maintain robust research practices that adhere to the highest ethical standards.

### Methodological challenges in multidisciplinary linkages must be addressed

There is a wide existing community of like-minded researchers generating insight and evidence from digital footprint data, comprising a variety of disciplines and approaches. While this is a strength of the field, it also serves as a barrier to sharing knowledge due to differences in terminology, methodologies and research priorities across disciplines. To address this, we must ensure that there are ongoing platforms to share learnings and avoid efforts being duplicated, such as the multidisciplinary and multisector Digital Footprints conference,^6^ or regular workshops hosted through the Turing Novel Data Linkages Interest Group.^4 5 7^

It is noteworthy that many methods will be specific to novel data sources, such as social media, sensors, geospatial sources and images. While there is an opportunity to share technical skills across domains and disciplines to capture contextual variations and learnings, it is important to recognise that procedures and solutions are likely to be bespoke to a given research question. Further, while many administrative and medical data sources are already routinely used in research, linking them with digital footprint data is innovative. Determining a glossary of terms is crucial; first, to promote consistent language and terminology, and second, to enable inclusivity by finding common grounds between domains and disciplines. This process should involve governmental bodies and the public sector, emphasising the need for a platform to communicate and catalogue these metadata.

Reproducibility is crucial for the sustainability and long-term value of novel data linkage research. The issue is complicated by the fact that the data are often commercial and cannot be shared in the public domain, hence, alternative ways to evaluate data quality and realise research transparency are needed. In addition, it is necessary to invest in training that can support both technical and ethical capabilities and support Early Career and Transdisciplinary Researchers in following these standards.

Finally, using linked datasets requires multidisciplinary and novel approaches that are often overlooked in the single-domain framework that drives academic publications and funding bids. Academics who develop research programmes in this field are considered pioneers, and these programmes face a known risk to project longevity and sustainability. This risk arises from the challenge of securing funding for unknown approaches, creating a circular problem: without funding, researchers may lose access to essential data, thereby jeopardising project continuation. As such, funders must shift from relying on discipline-specific reviewers to reward interdisciplinary novel data linkages more efficiently. Early Career Researchers in particular—balancing the demands of prestigious academic publishing with exploring new data sources and engaging in cross-sector research—require a supportive academic environment that values the multidisciplinary nature of novel data linkage research.

## Conclusion

In conclusion, we believe that harnessing the full potential of digital footprint data for the public good requires successful collaboration between policy-makers, industry organisations and academics. This discussion highlights the unique obstacles faced by each stakeholder group. We contribute a multiperspective overview of this topic, underscoring the need for policymakers to engage in digital footprint research, the importance of shared value in industry–academic partnerships and the role of ethical considerations and consumer trust in shaping data linkage. We propose practical solutions for the challenges that we have outlined.

However, we acknowledge that our focus on the UK context limits the applicability of our discussion to other countries. Variations in legislative frameworks (such as the GDPR in Europe or the Health Insurance Portability and Accountability Act in the USA), cultural norms and healthcare infrastructures significantly impact data sharing and linkage practices. This means our recommendations may not directly apply internationally, and it would be beneficial to further explore international perspectives in future work.

## Supplementary material

10.1136/bmjhci-2024-101119 online supplemental file 1
