# Supplementary material for: Harnessing digital footprint data for population health: a discussion on collaboration, challenges and opportunities in the UK
Source: BMJ Health Care Inform. 2024 Sep 28;31(1):e101119. doi: 10.1136/bmjhci-2024-101119 (PMC11448216; doi:10.1136/bmjhci-2024-101119)
Supplement: online supplemental file 1 [file bmjhci-31-1-s001.pdf]

Supplementary Material

Overview of *Turing Novel Data Linkages SIG* events

The *Turing Novel Data Linkages Special Interest Group* brings national and international multidisciplinary researchers and multi-sector communities together to explore opportunities for linking novel digital footprint data to health and wellbeing outcomes.[4] The group aims to build a community to work together on challenges related to these linkages (e.g., papers, grant proposals, research projects, responses to government consultations), acting as a vehicle to connect conversations and people as a network to promote novel work in this area.

So far, the special interest group has organised three unique events;[5,6,7] the conversations, presentations, and outputs of these events were used to inform this Discussion.

Event 1: Inaugural Novel Data Linkages for Health and Wellbeing.[5]

The first event – which took place on 26 October 2022 – brought together this new community at the Alan Turing Institute, London. Group members represented a range of organisations, including: UK and overseas academic institutions, industry, government related organisations, cross sector research organisation, and an educational charity. The event hosted a series of talks, panels, and discussion groups, culminating in the emergence of key themes and future actions as the drivers in the future success of data linkage.

Event 2: Digital Footprints Conference 2023.[6]

The inaugural Digital Footprints Conference – hosted at the University of Bristol on 11 May 2023 – brought together the growing multidisciplinary and multi-sector community to make connections through sharing experiences, knowledge, methods, successes, and failures. This one-day event included a keynote speech, flash talks, poster presentations, a panel discussion, and various other talks. Overall, this event highlighted the supportive community of like-minded researchers eager to share knowledge and encourage one another, and underscored the importance of broader engagement between industry, policymakers, and other stakeholders.[6]

Event 3: How can Digital Footprint Data be used for Public Benefit?[7]

This meeting of the special interest group took place on 30 November 2023, once again at the Alan Turing Institute, London. Around 40 delegates were in attendance, representing academic, industry and government organisations. Primarily, discussions and talks during the event focused on opportunities and barriers to collaboration between these respective groups.
